# Estimation of Nitrite—Nitric Oxide Derivative—In Horses with Intestinal Colic by ESR Spectroscopy

**DOI:** 10.3390/vetsci7040191

**Published:** 2020-11-29

**Authors:** Seid-Fatima Borunova, Nikolay Tkachev, Baylar Iolchiev, Zinaida Artyushina, Pavel Abramov, Marina Nikitina, Anastasia Silanteva, Neilia Khusnetdinova, Vladimir Serezhenkov

**Affiliations:** 1 The Russian State Center for Animal Feed and Drug Standardization and Quality (VGNKI), Zvenigorodskoye Shosse 5, 123022 Moscow, Russia; 2 Semenov Institute of Chemical Physics, Russian Academy of Sciences, 4 Kosygina Street, Building 1, 119991 Moscow, Russia; nitkachev@yandex.ru (N.T.); serezhenkov@yandex.ru (V.S.); 3 L.K. Ernst Federal Science Center for Animal Husbandry, Dubrovitsy 60, Podolsk Municipal District, 142132 Moscow, Russia; baylar1@yandex.ru (B.I.); 9790197@mail.ru (A.S.); 4 Skryabin, Academy of Veterinary Medicine and Biotechnology, 23, Scryabin Str., 109472 Moscow, Russia; artyshina.zinaida@yandex.ru (Z.A.); abramov_p@inbox.ru (P.A.); vet-doc@bk.ru (N.K.); 5 V.M. Gorbatov Federal Research Center for Food Systems of RAS, 26, Talalikhina Str., 109316 Moscow, Russia; nikitinama@yandex.ru

**Keywords:** ddinitrosil iron complexes, ESR-spectroscopy, horse, nitric oxide, nitrite, nitrosyl hemoglobin

## Abstract

Diseases of the gastrointestinal tract of horses are caused by many factors and have a complex pathogenesis. Developing effective methods of differential diagnostics is of high fundamental and applied importance. The pathogenesis of diseases of the digestive tract of horses accompanied by the development of inflammation and oxidative stress, can be associated with a lack of the nitrogen monoxide which controls many signaling pathways in the body. The level of the nitric oxide (NO) is involved in the regulation of the immune and nervous systems, the tone of all the blood vessels, and the courses of many pathological processes. The nitric oxide activates guanylate cyclase (sGC) and leads to vascular relaxation. The aim of this investigation was to study the metabolites of nitric oxide in horses suffered from intestinal diseases. The levels of nitric oxide in the blood serum of horses depending on their age and health state was studied. The concentration of nitrites in the blood serum of horses aged 6–25 years was 3.4 ± 4.2 μM, and in the young horses (1–5 years) the level of this indicator was 8.2 ± 5.4 μM. A sharp decrease in nitrite was observed in all the horses with intestinal diseases of 2 ± 0.9 μM, especially with tympanitic caecun of 0.6 ± 0.4 μM and with spasmodic colic of 1.8 ± 0.5 μM. The level of nitrosylhemoglobin HbNO in the blood of the diseased animals was higher than that in clinically healthy horses, regardless of age.

## 1. Introduction

The prevalence of diseases of the stomach and intestines in horses [1,2], the multi-factorial nature of their pathogenesis, as well as the complexity of differential diagnosis, demonstrate that this topic—which requires further study—is of high practical and theoretical relevance.

One of the main characteristics of gastrointestinal diseases in horses is the frequent development of endotoxic shock. Almost all diseases of the intestine of a horse cause a change in the composition and the quantitative ratios of its microflora, a decrease in the pH of the intestinal contents. The destruction of Gram-negative bacteria leads to the release of endotoxins. The endotoxins absorbed by the intestinal wall bind to specific CD14 receptors on the endothelial cells of blood vessels, dendrite cells, monocytes and macrophages. Through CD14 receptors, the endotoxins bind to toll-like receptors (TLR2, TLR4) and mitogen-activated kinases [3,4]. In other words, the diseases of the gastrointestinal apparatus of horses are of both acute and chronic nature, but in both cases oxidative stress reactions are involved. The result of oxidative stress activation is due to the hyperproduction of reactive oxygen species [5]. In the process of pathogenesis [6,7], the level of nitric oxide (NO) required for normal functioning of the vascular endothelium of the intestinal mucosa and sphincters may decrease. To account for the reduced level of NO in the development of oxidative stress, the concept of bioavailability of NO has been introduced.

In this respect, the role of microflora in the metabolism of nitro compounds (nitrites, nitrates and NO) is profound in the development of equine gastrointestinal diseases [8,9]. Numerous investigations have demonstrated the presence of bacterial products and pro-inflammatory cytokines as iNOS inducers in the gastrointestinal tract [10,11,12,13]. High levels of nitric oxide (NO) can be directly toxic to cells, causing apoptosis and necrosis of the enterocytes, disruption of the intestinal barrier which contributes to bacterial translocation, decreased motility and diarrhea [14]. Nevertheless, NO can exert a protective effect: an increase in the blood flow in the intestinal wall and the secretion of mucus, and can contribute to an increase in the antibacterial response [15].

Nitrogen monoxide (NO) takes part in the work of the immune and nervous systems, its level directly determines the tone of all vessels and the course of many pathological processes. NO is synthesized from L-arginine by the action of NO synthase (NOS). One of the most important functions of NO is vascular relaxation via activation of the heme-containing enzyme soluble guanylate cyclase (sGC). Specific physicochemical properties of this molecule determine the method of its transportation: in the form of nitrosyl complexes with heme iron, nitrosothiols nitrosocysteine (RSNO), and also high- and low-molecular weight complexes of non-heme iron with thiol ligands (glutathione, cysteine)—so-called “2.03 complexes” [16,17]. The formation of both dinitrosyl complexes of binding iron (II) thiol groups with NO (DNIC) and nitrosothiols of proteins represents a form of post-translational regulation of their activity [18]. This method of protein modification is selective, reversible and stabilizes the nitric oxide (NO) in a unique bioactive form.

Measurement of the concentration of nitrates and nitrites using the Griess reagent is widely practiced to quantify the severity of the inflammatory process [19]. This approach is complicated due to the methodological difficulties associated with the measurement, which leads to a variation of data up to 10,000 times from 1 nM to 7 μM in tissues and fluids [20]. In diseases of the gastrointestinal tract in the serum of horses, the nitrite and nitrates were determined by the Griess method [21,22]. Despite the obvious clinical indicators of gastrointestinal diseases in animals, the assessment of the total level of nitrates and nitrites [23] did not allow for differentiating the physiologic from pathologic state, even when many samples of animals were included in the investigation. Both with the pathology of the gastrointestinal tract and in the physiologic state, the measurement of the product of NO metabolization—nitrite [24,25]—is the most accurate assessment of its level in the body.

Therefore, the aim of our work—based on the analysis of literature data on the possible association of nitric oxide levels in the blood serum of horses with symptoms of colic, and gastrointestinal tract pathology in general—was the measurement of the NO-nitrite metabolite level by electron spin resonance spectroscopy using spin traps [26,27].

## 2. Material and Methods

### 2.1. Legal Requirements

The protocols of experiments were determined by the Committee for Animal Care and Use of the Moscow State Academy of Veterinary Medicine and Biotechnology in accordance with Decision No. 80 of the Council of the Eurasian Economic Commission of 10 November 2017 “On Approval of the Rules for Organization of Laboratory Testing during Veterinary Control (Supervision)”. The protocols of experiments (Number of report 3, 11 February 2020).

### 2.2. Animals

The object of the investigation was horses, of which 38 animals were studied altogether. In the clinical study, 8 were diagnosed with: spasmodic colic, impaction of the left ventral colon, large colon displacement, seasonal colic, tympanitic caecum or colitis. NO forms stable complexes with iron of hemoglobin that can be detected directly in blood samples by the EPR method. To determine the NO in serum, we used the spin trap method based on the use of dithiocarbamate and iron (II).

For the study, blood was taken from the jugular vein into heparin tubes and centrifuged for 10 min in a centrifuge—model CH80-2S “Armed” at the speed of 3000 rpm to precipitate erythrocytes. After that, serum samples were stored in liquid nitrogen (−196 °C). To measure the concentration of nitrosylhemoglobin, whole blood (without heparin) was frozen in plastic containers of cylindrical shape and with a length of 35 mm and a diameter of 4 mm. EPR signals were recorded on a spectrometer Bruker ECS-106 (Germany).

### 2.3. Determining Nitric Oxide (NO)

The method of determining NO used in this work is based on the reaction of nitrosothiol formation—nitrosocysteine (RSNO) formation in an acidic medium (pH = 3.5) from the nitrite anion NO2- and cysteine hydrochloride. Then, nitrosocysteine, in the presence of iron (2+) N-methyl-D, L-glucamine dithiocarbamate (MGD), forms a water-soluble paramagnetic mononitrosyl iron complex MNIC MGD-Fe-NO. The determination of the nitrite anion NO2- was carried out as follows: serum proteins of a weight greater than 30 kDa after defrosting were removed by filtration through a Microcon 30 kD filter (Millipore Corporation, USA) for 20 min at 14,500 rpm in a Mini Spin plus centrifuge, Eppendorf. The developed method makes it possible, first, to control the effect of serum proteins on the reaction of the MNIC MGD formation and secondly, to take into account the contribution of high molecular weight RSNO and DNIC as sources of NO. To 50 μL of cysteine at a concentration of 400 mM, 10–120 μL of serum was added after filtration. The pH of the solution was adjusted to 3.5 by adding 0.01 mM HCl. After 5 min, 50 μL of 40 mM iron (II) sulfate, 200 μL of 400 mM buffer (Tris or Hepes) and 200 μL of MGD with a concentration of 250 mM were added. Then the pH of the solution was raised to 7.6 with a 0.06% NaOH solution. Under these conditions, the MNIC MGD-Fe-NO is formed.

### 2.4. Data Analysis

**Figure 1:**
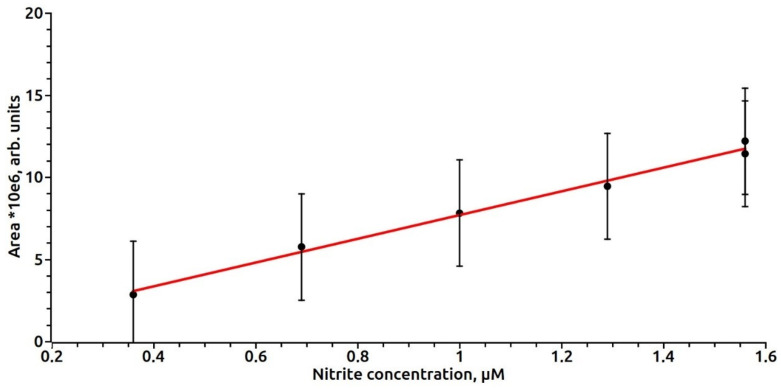
The dependence of the intensity of the EPR signal on the nitrite concentration in the sample after removal of serum proteins.

To construct a calibration curve, a sodium nitrite solution of a concentration of 480 μM in the various volumes (2–40 μL) was added to 50 μL of cysteine hydrochloride with a concentration of 400 mM, the pH of the solution was adjusted to 3.5 by adding 0.01 mm HCl. After 5 min, 50 μL of 40 mM ferrous sulfate was added, 200 μL of 200 mM (Tris or Hepes), 200 μL of 250 mM MGD, the pH was adjusted to 7.6 with 0.06% NaOH solution. After 10 min, the ESR spectrum of the MNIC MGD-Fe-NO was recorded. The concentration of nitrite in the sample was estimated by the method of double integration and construction of a calibration curve (Figure 1). The dependence of the EPR signal intensity on the nitrite concentration in the range of 0.3–1.6 µM in the sample after the removal of serum proteins was approximated by a linear relationship (C) = 0.134 S − 0.024. Spearman’s correlation coefficient was estimated as r = 0.984. Calculations of the EPR signal areas were performed using the Bruker ECS-106 EPR spectrometer software.

### 2.5. Statistical Analysis

To estimate the degree of difference, analysis of the distribution form was used (Kolmogorov–Smirnov test) and comparison of medians and means (Mann–Whitney U-test and Student’s *t*-test), respectively.

## 3. Results

In case of measurements of endogenous nitrite in the presence of serum proteins, the EPR signal of MNIC MGD-Fe-NO was reduced by 24–57%. The nitrosyl groups of the high molecular weight RSNO, in case of the presence of RSNO in serum, can be oxidized to nitrite during long-term manipulations with samples, and this can also affect the variability of the data. We believe that the low molecular weight RSNOs that are available do not make a significant contribution to the measurement of nitrite. To catalyze their decomposition and the formation of nitrite, copper Cu+ must be present in the alkaline (pH = 10.5) solution or the medium. In our studies, the reduction of nitrite and its inclusion in the MNIC MGD-Fe-NO is carried out at pH = 3.5. In case of the formation of RSNO from nitrite and glutathione or serum cysteine, the latter can be in present in low concentrations, then, after addition of a trap MGD-Fe form, the paramagnetic MNIC MGD-Fe-NO with the participation of the nitrosyl group of the newly RSNO is formed. The addition of a trap at pH = 7.4 to the serum of diseased and healthy animals did not cause the formation of the paramagnetic complex MNIC MGD-Fe-NO (signal-to-noise ratio = 1), which may indicate the absence of significant amounts of RSNO and DNIC in the serum. According to our estimates, this level in total did not to exceed 30 nM.

**Figure 2:**
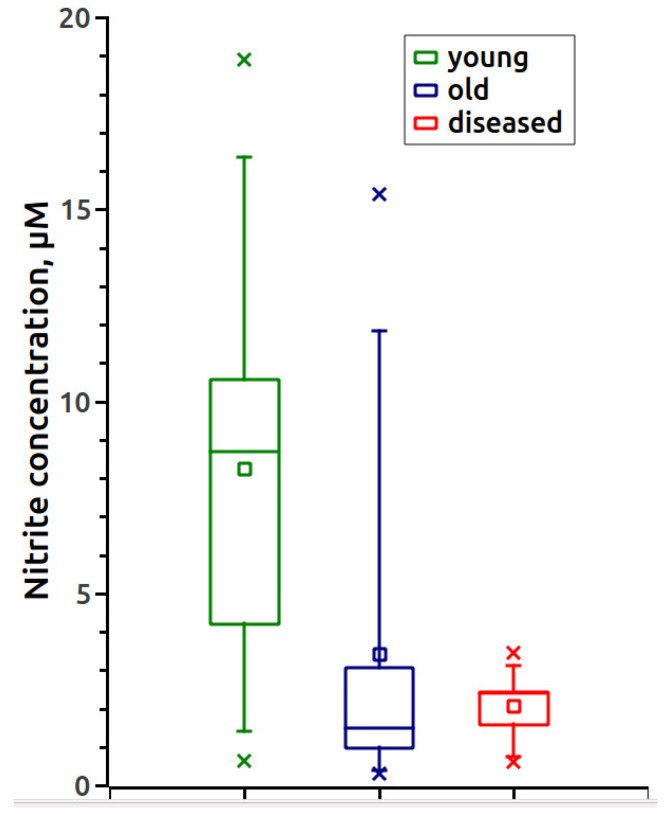
Concentration of serum nitrite in different groups of horses.

**Figure 3:**
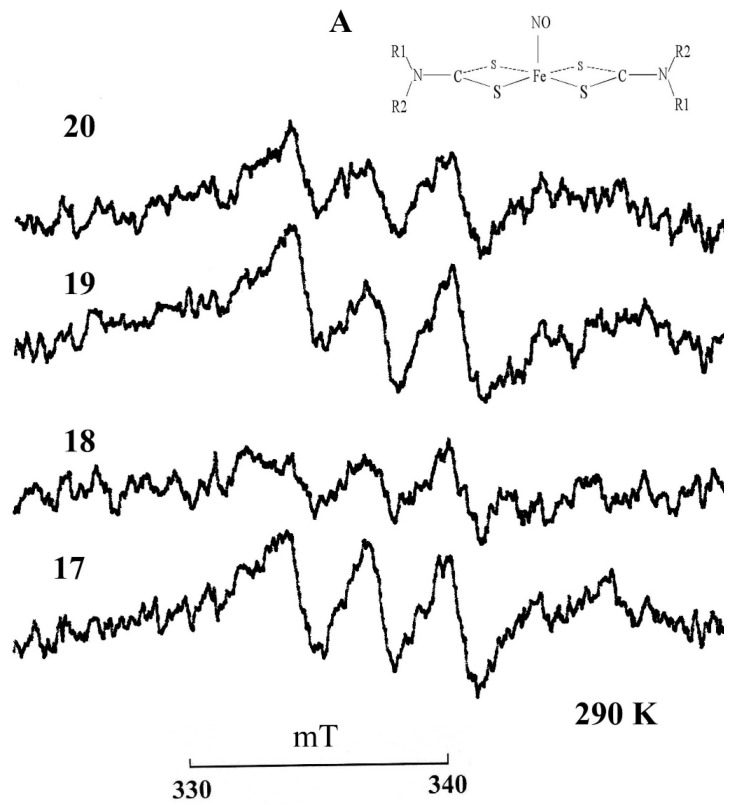
EPR spectra of MGD complex with iron (2+) and nitric oxide MNIC MGD-FeNO in the horse serum: No.17—left ventral colon obstruction, No.18—tympanic caecum, No.19—cecal impactioin, No.20—spasmodic colic. Insert A is the structural formula of the paramagnetic complex MNIC MGD-FeNO. R1 = CH_3_, R2 = CH_2_(CHOH)_2_CH_2_OH–N-metil-D,L-glucamine. Registration conditions: X-range; center field 344 mT; sweep width 10 mT; modulation amplitude 0.05 mT ; microwave power 20 mW. Amplifcation, 1 × 10^5^. T= 293 K.

Using the constructed calibration curve, we measured the nitrite content in the serum samples of animals, which is shown in Table 1 and Figure 2. Figure 3 shows the EPR spectra of the MNIC MGD-Fe-NO from horses with a gastrointestinal diseases.

Table 1 shows the results of measurements of nitrite in the serum of healthy animals of the two aged groups: 1–5 years and 6–25 years, as well as eight animals with pathology.

The results of the statistical analysis of the obtained data are shown in Table 2. The statistical analysis showed significant differences in all parameters studied for aged and diseased horses compared to young ones (*p* = 0.0484 and 0.0108; 0.0074 and 0.0391, respectively), whereas the differences between aged and diseased samples were not significant (*p* = 0.6547 and 0.2500).

## 4. Discussion

Nitric oxide (NO) should be considered as one of the most important factors for protection of the gastrointestinal mucosa. The blockade of NO formation affects the secretory and regenerative functions of the stomach. The role of NO as a neurotransmitter in non-adrenergic/non-cholinergic neurons (NANC neurons), which, along with the choline- and noradrenergic conductors of the autonomic nervous system, constitute a third of the autonomic nervous system [28,29]. The activity of K+ channels in conducting of the impulses in neurons strongly depends on the degree of nitrosylation of cysteines of the channel-forming protein [30]. Nitrosylation of cysteines in four of five protein fragments slows down the conduction of impulses in neurons. Horses have three autonomous centers for regulation of peristalsis associated with the interstitial cells of Cajal: the gastroduodenal center, the ileo-celiac center and the pelvic flexure center [31]. In addition to NO, the autonomic intestinal nervous system uses vasoactive intestinal peptide (VIP) and substance P as neurotransmitters. It was established that nitrogen oxide has a significant role in the activation the synthesis of bicarbonates, regulating blood flow and gastrointestinal motility. The participation of NO in the pathological processes occurring in the large intestine has been shown previously [32,33]. In particular, experimental colitis is accompanied by a decrease in the expression of nNOS and impaired colon relaxation. These changes may contribute to impaired motility and intestinal absorption. According to recent data, an increased total level of nitrates and nitrites characterizes the course of inflammatory processes in the body, as a result of the increased iNOS activity. Oxyhemoglobin and oxymyoglobin are able to convert some of the excess NO to nitrate. It is well known that in this case, oxidative stress develops in cells and tissues and processes are initiated with the participation of the reactive oxygen species. As a result, effective NO is transformed to active forms of nitrogen and nitrates. Tsikas et al. [20,25] found that at pathology, the level of nitrates in human serum increases and the level of nitrites decreases in relation to the norm.

To explain the reduced level of NO in the development of oxidative stress, the concept of bioavailability of NO has been introduced. This means that NO production is high, but its action as a messenger is small. At certain stages of the disease there is a deficit of NO and thus, the reduced bioavailability of NO causes a violation of the functions of the gastrointestinal tract in horses. Metronidazole has been used to treat horses and people with gastrointestinal diseases. Why is this so? Mason et al. [34] demonstrated the formation of nitric oxide from the nitro group of metronidazole in the form of the MNIC and DNIC complexes in the presence of iron and cysteine. The NSAID NO-aspirin (NCX-4016), which releases NO, has a similar effect. Slivka et al. [7] demonstrated inhibition of the function of the Oddi's spincter by the nitric oxide donor S-nitroso-acetylcysteine. Experiments on rodent colitis models have shown a positive effect of fatty acid nitrates [35]. Lee et al. [36] examined the healing of ulcers and changes in the microcirculation caused by the synthesis of nitric oxide (NOS) induced by beta-dexate hydrochloride (BHB).

**Figure 4:**
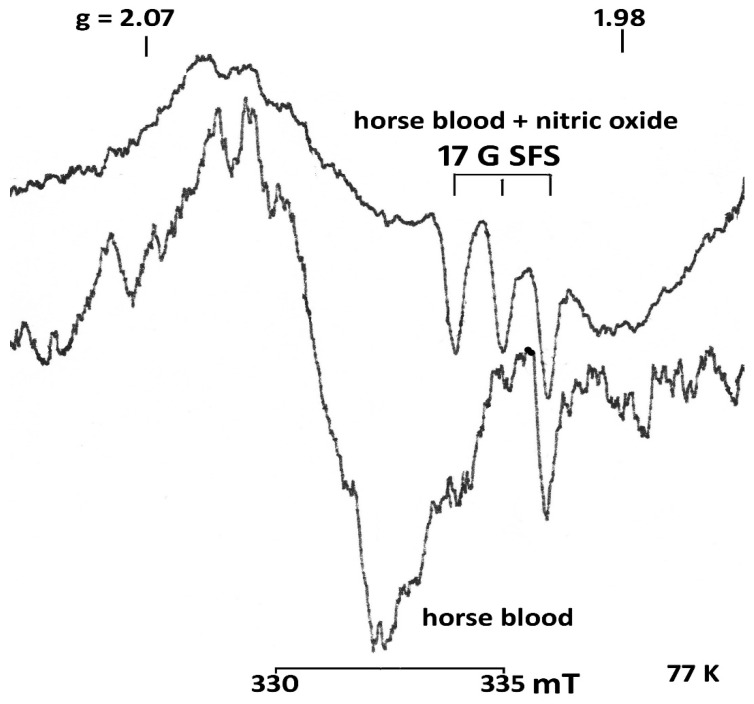
EPR spectra of blood of a healthy horse and blood treated with gaseous nitric oxide (horse blood + nitric oxide (NO)). The blood of a healthy horse contains nitrosylhemoglobin (0.2 ± 0.9 μM). The blood was treated with nitric oxide in the concentration 12.3 ± 0.3 μM. SFS—super fine structure Registration conditions as above. Amplifcation horse blood + nitric oxide (NO)−2 × 105, 4 scans, horse blood −1 × 105, 1 scan. T = 77 K.

The data obtained in this study demonstrate that the concentration of nitrite (Table 1 and Figure 3) in the serum of horses aged 6–25 years was 2.4 times lower than in young animals. An acute decrease in nitrite was observed in all horses with intestinal diseases, especially those with tympanitic caecum and spasmodic colic. The level of nitrosylhemoglobin (Table 1 and Figure 4) is lower in the whole blood of the two age groups of healthy horses compared to the diseased animals.

**Figure 5:**
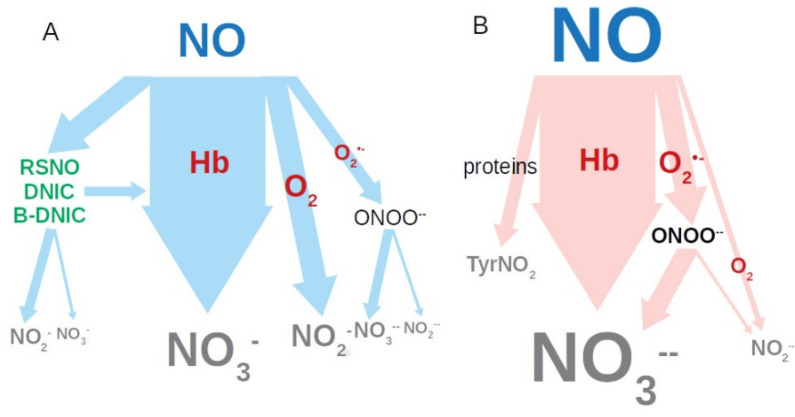
Nitric oxide metabolizing pathways: (**A**) normal; (**B**) with oxidative stress. RSNO: nitrosyl thiols; DNIC: iron (II) thiol groups with NO; B-DNIC: binuclear DNIC.

These facts are important for further studies of the diagnostic and prognostic value of nitric oxide as a biomarker for the regulation of intestinal peristalsis in normal and pathological conditions. It begs the question as to why serum nitrite levels are lower in aged horses and those with gastrointestinal diseases compared to healthy ones, even though NO is synthesized in greater quantities? The following is proposed as an explanation. Both in physiological and pathological states, the main mechanism for the removal of excess NO is its transformation into nitrate by the oxygenated heme of hemoglobin (Figure 5A).

At the same time, part of NO manages to react with oxygen to form nitrite. The transformation of NO can also occur through interaction with the superoxide anion radical formed during inflammation, with the formation of peroxynitrite. At physiological pH 7.4, it is unstable, easily protonated and decomposed leading to the formation of both nitrates 70% and 30% nitrites. Thus, in a state of inflammation and oxidative stress, pathways for metabolizing NO are shifted towards the formation of nitrate (Figure 5B). It is also possible that NO is partially spent on the formation of DNIC [37] and an uncontrolled nitrosylation of protein thiols, in addition to the formation of nitrotyrosine in proteins [38]. However, the contribution of the above reactions is less significant than formation of nitrate.

There is reason to believe that in the serum of aged and diseased horses, beside nitrites, there is also a pool of low molecular weight RSNO with a mass below 30 kD and a pool of DNIC (or binuclear DNIC: B-DNIC) with thiyl ligands. Normally, both serum RSNO and DNIC (or B-DNIC binuclear DNIC), are present in a higher concentration as donors and transporters of NO. According to Tsikas et al. [25], in normal human serum, the limit of RSNO can reach 200 nM. (25) The hypothesis of the presence of DNIC or B-DNIC in the serum of horses proposed by us does not contradict the available data in the literature. The work of Hickok et al. [37] provides data on the mutual relationship in cells and tissues of RSNO and DNIC, the latter is four to eight or 30 times greater. The level of nitrosylhemoglobin in the serum of healthy, aged, and diseased horses (Table 1), measured by us, ranges from 0.1 ± 0.1 μM to 0.3 ± 0.1 μM, respectively. Thus, this suggests that the limiting level of RSNO, DNIC or B-DNIC in horse serum is 200–300 nM. It was shown that with colic of the gastrointestinal tract the level of the main cellular antioxidant glutathione decreases, which may lead to an increase in the role of hemoglobin in NO metabolism [39]. Thus, our data on the reduction of nitrite in serum may indicate a decrease in the both transport and regulatory forms of NO: RSNO, DNIC or B-DNIC in the serum of horses.

## 5. Conclusions

The results of the study show that in the state of inflammation and oxidative stress, the pathways of NO metabolism shift towards the formation of nitrates. It is also possible that NO is partially consumed for the formation of DNIC, the uncontrolled nitrosylation of protein thiols, and the formation of nitrothyrosine in proteins. Studies on the assessment of NO and its metabolites in the blood should continue to distinguish a more complete picture of the processes occurring in the complex symptoms of colic in horses. The spin trap technique and EPR spectroscopy, applied by us, allows one to take into account NO in both RSNO and DNIC. For a more correct assessment of the inflammatory process, it is necessary to study the NO-producing activity in the tissues and fluids of the zone of inflammation.

## Figures and Tables

**Table 1:** Concentration of nitrite in serum and nitrosylhemoglobin HbNO in whole horse blood.

Animal Groups	Nitrosylhemoglobin Concentration, μM, Mean ± SD	Serum Nitrite Concentration μM, Mean ± SD
Healthy horses Aged 1 to 5 years, *n* = 11	0.1 ± 0.1	min 0.6 ± 0.3 max 18.9 ± 0.6
Healthy horses Aged 6 to 25 years, *n* = 19	0.15 ± 0.1	min 0.30 ± 0.2 max 15.4 ± 0.5
Ventral colon obstruction, 13.5 years	*	2.4 ± 0.7
Tympanic caecum, 7 years	*	0.6 ± 0.4
Cecal impaction, 14 years	*	2.4 ± 0.7
Large colon displacement, 8 years	0.3 ± 0.1	2.4 ± 1.3
Colitis, 18 years	*	2.5 ± 0.6
Seasonal colic of pelvic flexure, 3.5 years	*	2.3 ± 0.6
Spasmodic colic 6.5 years	0.2 ± 0.1	1.8 ± 0.5
Recurrent colic 3 years	*	6.1 ± 2.1

* Nitrosylhemoglobin level could not be detected (below 0.1 μM).

**Table 2:** Statistical data.

	Young 1–5 Years	Aged 6–25 Years	Diseased
Mean ± SD, μM	8.24 ± 5.42	3.42 ± 4.22	2.07 ± 0.90
Median, μM	8.7	1.5	2.4
Distribution normality Shapiro–Wilk test, *p*	0.79	7 × 10^−5^	0.4274
Distribution function Kolmogorov–Smirnov test, *p*	0.0484	0.007 ^a^	0.654
Median Mann–Whitney U-test, *p*	0.010	0.039 ^b^	0.250
Mean Student’s *t*-test, *p*	0.021	0.003 ^c^	0.1990

^a,b,c^: indicates strings with different superscripts that are statistically different.
